# Solubility of Structurally Complicated Materials: 3. Hair

**DOI:** 10.1100/tsw.2009.27

**Published:** 2009-04-27

**Authors:** Ari L. Horvath

**Affiliations:** ICI Chemicals and Polymers Limited, The Heath, Runcorn, Cheshire, UK

**Keywords:** solubility, structurally complicated materials, hair

## Abstract

Hair is composed of proteins, lipids, water, and small amounts of trace elements. All proteins in animal and human bodies are built from permutations of amino acid molecules in a polypeptide string. The polypeptide chains of protein keratin are organized into filaments in hair cells. Hair is one of the most difficult proteins to digest or solubilize. Among the most common dissolving procedures for hair are acidic, alkaline, and enzymatic hydrolysis. For the analysis of hair, the solid samples are transferred by solubilization via digestion into a liquid phase. Small molecular solvents and molecules with hydrophobic groups appear to have higher affinity for hair. A good solvent attacks the disulfide bonds between cystine molecules and hydrates the hair shaft. Consequently, the hair becomes a jelly-like mass.

### INTRODUCTION

Design and prediction of structurally complicated materials is one of the most challenging problems in computational sciences. Developments in many body calculations for condensed matter have been primarily empirical.

It has long been a dream of chemists and physicists to be able to explain the properties of solid mixtures using only knowledge of the properties of their constituent molecules. Despite the advent of quantum mechanics, however, the technique to calculate and predict structural properties accurately has not been realized.

A program was undertaken to compile, evaluate, and consolidate all relevant original test data on the solubility of structurally complicated materials published during the period 1970–2005 inclusive. This paper contains the accumulated information and presents 10 years of effort. The solubility of structurally complicated materials is presented in several papers in this series, divided as follows: wood[1], bone[2], hair (this paper), skin, etc.

Whether a structurally complicated material (solute) consisting of several components is soluble, partially soluble, or insoluble depends on several properties of both the solute and the solvent, or solvent blends. The state of the solute, i.e., whether it is solid, liquid, or gas, has a major influence on the solubility, in addition to the physical properties of the solute and solvent. Many of these parameters and their effects on the solubility or insolubility are outside the scope of this paper.

This paper briefly summarizes the findings of the extensive literature survey conducted to study the methods reported for measuring the solubility of hair. It is not within the scope of this paper to describe, in detail, however, the various methods and techniques reported in the literature. The reader is directed to the references listed at the end of this paper for more detailed information regarding the reported experiments.

There are no compilations either by individuals or by organized societies that are currently engaged in the systematic collection of solubility data of structurally complicated materials. Currently, the International Union of Pure and Applied Chemistry (IUPAC) is engaged in the systematic compilation and critical evaluation of the solubility of pure solids, liquids, and gases. This is an ongoing compilation for the Solubility Data Project. The objective of the project is to collect and evaluate all relevant data and present them in tabular form. A detailed description of contents and of the methods of data selection is given in a series of IUPAC volumes, approaching 100 in size (http://www.iupac.org/publications/sds/volumes.html). Solubility data are so widely scattered throughout the technical literature that compilations are an indispensable aid for workers in chemical industries and many universities.

Among the useful information on the solubility of hair, there are three areas that often cover the experimental results in the published literature. These are

1. Forensic science (detecting drugs of abuse)
2. Medical science (removal of undesirable hair in clinical cases)
3. Cosmetics (research for chemical depilatories)

The compiler assumes that readers of this article have some knowledge of organic chemistry and biochemistry. The author has relied on the open literature, personal communication from colleagues, and his experience in the determination of composition of hair.

Some relevant articles are undoubtedly inadvertently omitted from this work. For this, apologies are offered and reprints of the omitted material, with appropriate references, are respectfully requested.

## THEORY

### Structure and Properties

During the course of biological evolution, the building-block compounds performed specific cell functions. Biomolecules serve as building blocks of cells. All living matter is composed of cells. All cells arise from pre-existing cells. Deoxyribonucleic acid (DNA) is the most important molecule in living cells.

Simple organic molecules present in living material include monosaccharides, amino acids, fatty acids, glycerol, and aromatic bases. These monomers act as building blocks for the synthesis of larger macromolecules, called polymers, by condensation reactions.

When many amino acids are joined together by peptide bonds, a polypeptide or protein is formed. Proteins are macromolecules consisting of one or more polypeptide chains. They comprise 50% of the total dry mass of animal cells.

Hair is a biological material consisting of polypeptide chains of keratin arranged into filaments. In most mammals, hair increases the sensitivity of the skin surface and forms an insulating and protective coat. Hair reduces heat loss from the body and often provides camouflage. For humans, hair is important only for personal adornment and display[3]. In lower animals (e.g., insects), hairs (whiskers) have a sensory function. Some stems, leaves, and plants also possess hairs on their roots.

Hair is a complex tissue and grows from the hair follicle embedded in the inner layer (dermis) in the skin where the germination center is formed by matrix cells that are in active build-up to layers of the hair shaft, including the cuticle, cortex, and medulla. The cortex forms the bulk of the hair shaft and is located immediately beneath the cuticle. The medulla is the innermost region of hair and consists of scattered cells and hollow space.

Human hair is not homogeneous. In the outer layers of hair, the surface composition may vary rapidly[4].

The growth of human hair in all body sites is cyclic. Hair growth rate depends on anatomical region, age, gender, ethnicity, and interindividual variability. Hair growth varies between 0.7 and 3.6 cm/month. On average, the rate is 0.8–1.4 cm/month[5]. A mean of 1 cm/month is generally accepted[6]. Different phases of the hair cycle include:

- Anagen growing stage, 4–6 years
- Catagen transition stage, 2–5 weeks
- Telogen resting stage, 4–6 months

Whether hairs are in anagen or telogen phase at the time of removal is important because only anagen hairs are particularly sensitive to chemical, cytostatic, physical, hormonal, infectious, or inflammatory invasion.

As noted above, hair is an epidermal outgrowth from a hair follicle. Hair is made up of many fibrils and each fibril, in turn, is made up of sets of even smaller fibers that are twisted around each other in ropelike arrays. That is, hair fibers consist of multistranded ropes or cables in which each strand is an α-helical polypeptide chain and the strands are supercoiled around each other. The three-strand arrangement of the polypeptide chains of hair is held together by covalent cross-links between the adjacent polypeptides provided by cystine residues. The cystine content determines the softness or hardness of the α-keratins. Keratin is a general term used to describe aggregates of protein with low or high sulfur content. The external layers of skin, wool, feathers, and hair are composed of softer keratins[7].

Hair is composed of approximately 65–95% protein, 1–9% lipids, melanins (complex polymers derived from tyrosine, a nonessential amino acid), small quantities of polysaccharides, 3–5% water, and 0.25–0.95% trace elements. The proteins are synthesized in the keratogenous zone of the hair follicle as matrix cells, and they move upward from the hair bulb to form layers of the hair shaft. The cuticle, cortex, and medulla are comprised largely of keratin, although it is structurally different in each layer.

Lipids are water-insoluble, oily or greasy organic substances that are extractable from cells and tissues by nonpolar solvents, such as chloroform or ether. The most abundant kinds of lipids are the fats or triacylglycerols, which are the most important form for chemical energy storage. Simple lipids are made up of three chains of fatty acids attached by an ester linkage to a molecule of glycerol to form a triglyceride, a storage form of fat. Lipids also contain other elements; in the phospholipid molecules, one fatty acid is replaced by a phosphate molecule plus a water-soluble molecule.

The hair surface is hydrophobic and the hair core is hygroscopic. Color is determined by the amount of melanin pigment in hair. Melanin is a brownish-black pigment of the skin, hair, and eyes. It is formed from tyrosine, $C_6H_4OHCH_2CHNH_2COOH$, a nonessential amino acid. Tyrosine is soluble in water, slightly soluble in alcohol, and insoluble in ether. The basic elemental composition of hair[8] (IRCP, 1975) is

- 49.0% carbon
- 14.5% nitrogen
- 30.0% oxygen
- 3.0% sulfur
- Trace elements barium, calcium, chromium, copper, iron, manganese, nickel, lead, titanium, zinc

Hair is a poor electrical and thermal conductor. Some morphological properties of ethnic head hair types are listed in Table 1[9]. Hair densities vary from 1.3 to 1.47 g/cm$^3$. Hair diameters have a mean and standard deviation of 67.1 ± 12.0 μm.

Protein keratin is made up of chains of amino acids, especially arginine, cystine, and serine. As noted above, the softer keratins occur in the external layers of skin, hair, wool, and feathers, while the harder types constitute nails, claws, and hoofs. The chemical analysis and the weight of amino acids in wool, for example, are listed in Table 2[10].

**TABLE 1**
**Morphological Properties of Ethnic Hair Types**

| Hair | Africoid | Caucasoid | Mongoloid |
|---|---|---|---|
| Diameter (µm) | 76.10 ± 15.83 | 79.17 ± 11.27 | 95.53 ± 10.35 |
| Fibrous protein (mg/0.1 g hair) | 8.6 ± 0.8 | 9.2 ± 0.7 | 14.3 ± 0.8 |
| Matrix protein (mg/0.1 g hair) | 46.0 ± 2.7 | 31.9 ± 1.3 | 32.9 ± 2.7 |

From Kidwell and Blank[9].

**TABLE 2**
**Weight of Amino Acids in Wool**

| No. | Amino Acid | Formula | Weight (%) |
|---|---|---|---|
| 1 | Alanine | $C_3H_7O_2N$ | 4 |
| 2 | Arginine | $C_6H_{14}O_2N_4$ | 19 |
| 3 | Aspartic acid | $C_4H_7O_4N$ | 4 |
| 4 | Cystine | $C_6H_{12}O_4N_2S_2$ | 7 |
| 5 | Glutamic acid | $C_5H_9O_4N$ | 9 |
| 6 | Glycine | $C_2H_5O_2N$ | 7 |
| 7 | Histidine | $C_6H_9O_2N_3$ | 2 |
| 8 | Isoleucine | $C_6H_{13}O_2N$ | 2 |
| 9 | Leucine | $C_6H_{13}O_2N$ | 6 |
| 10 | Lysine | $C_6H_{14}O_2N_2$ | 4 |
| 11 | Methionine | $C_5H_{11}O_2NS$ | 0.3 |
| 12 | Phenylalanine | $C_9H_{11}O_2N$ | 2 |
| 13 | Prolyne | $C_5H_9O_2N$ | 5 |
| 14 | Serine | $C_3H_7O_3N$ | 9 |
| 15 | Threonine | $C_4H_9O_3N$ | 5 |
| 16 | Tryptophan | $C_{11}H_{12}O_2N_2$ | 1 |
| 17 | Tyrosine | $C_9H_{11}O_3N$ | 3 |
| 18 | Valine | $C_5H_{11}O_2N$ | 4 |
| | | Total | 93.3 |

From Atkins[10].

The polypeptide chains of keratin are organized into filaments in hair cells. α-Keratin gives a characteristic diffraction pattern. This pattern shows that hair and wool possess a repeating structural unit along the long axis of the hair. Consequently, the polypeptide chains in the fibrous proteins are coiled in some regular way.

Four kinds of fibrous proteins (α-, β-keratins, collagen, and elastin) are responsible for the structure of cells, tissues, and organisms. The chief structural component of hair is protein keratin.

Keratin in the cortex comprises 85% or more of the mass of the hair shaft. Cortical keratin is composed of two types of structural proteins: matrix and fibrous. Matrix proteins have a high sulfur content and contain polypeptides with a molecular weight of approximately 10–28 kDa. The sulfur content of human hair is high compared with other animal species. Fibrous proteins are embedded in matrix proteins and are characterized by a low sulfur content. Matrix proteins have a nonhelical structure and are readily soluble at pH 4.5 in 0.5 *M* KCI, whereas fibrous proteins exhibit a helical structure and are insoluble in the same solvent[11].

Two types of proteins can be classified according to their structure/shapes: fibrous and globular. The fibrous proteins are long and stringy molecules with the polypeptide chains extended along one axis rather than folded into a globular shape. In globular proteins, the polypeptide chains are tightly folded into compact spherical or globular shapes. Nearly all enzymes, antibodies, and blood transport proteins are globular proteins. The hydrophilic or polar groups of globular proteins are exposed on the outer surface and therefore are soluble in water, e.g., serum albumin.

Fibrous proteins are the major components of the outer layer of skin, nail, hair, feathers, and horns. They are also the major organic components of connective tissues, including tendons, cartilage, bone, and the deeper layers of skin. α-Keratins constitute almost the entire dry weight of hair, wool, feathers, nails, claws, quills, scales, horns, hooves, tortoise shells, and part of the outer layer of skin. They are similar in their amino acid composition and spatial arrangement of their polypeptide chains.

One of the characteristic differences between the fibrous and globular proteins is their solubility in water. α-Keratins are rich in amino acids that have hydrophobic or water-insoluble groups, such as alanine, phenylalanine, valine, isoleucine, and methionine. α-Keratins are insoluble in water and organic solvents at ambient temperature. The molecules contain both acidic and basic groups and are thus amphoteric.

Both aspartic and glutamic acids are amino acid residues in keratin. The basic amino acid residues are arginine, histidine, and lysine. More basic groups than acidic groups are in keratin. Among the reactive groups are free carboxyl, amide, aliphatic and phenolic hydroxyls, and sulfhydryl groups.

Cystine is the most reactive amino acid residue in keratin. This is explained by the presence of disulfide bonds that can be oxidized or reduced. These reactions rearrange the structure of keratin and affect the physicochemical properties of hair.

Water molecules that approach peptide bonds may form water-polypeptide bonds based on rapid hydrogen bond exchanges between water and carboxyl and amino groups present in keratin.

Experimental results show that the different ethnic origins of head hair samples constitute different amounts of amino acids, see Table 3[12].

There are some structural similarities between hair of different colors, ethnic origin, and body region. Differences between scalp hair and hair from other anatomical regions are not yet fully investigated.

### Analysis

Several biological materials, e.g., blood, urine, saliva, sweat, and hair, have been proposed for the determination of drugs of abuse. Hair sample analysis provides several advantages over the analysis of blood, urine, and saliva samples because of easier sampling, sample storage, and detection of longer-term and less recent usage. The analysis of different longitudinal sections of head hair samples can give information on the history of the hair exposure to the average distribution of trace elements and drugs in a considerably large mass of hair.

Hair is best allocated from the area at the back of the head, called the vertex posterior. This area has less variability in hair growth rate than other areas. Moreover, the hair is less subject to age- and sex-related influences. There is, however, a relatively large individual variability. The sample size collected should be at least 100–250 mg.

For the analysis of hair, the solid samples are transferred into a liquid phase and then examined like a blood or urine sample.

Most of the analytical methods applied in hair analysis of a number of drugs are based on gas chromatography mass spectrometry (GC-MS), high-resolution mass spectrometry (HRMS), high-performance liquid chromatography (HPLC), headspace solid-phase microextraction (HS-SPME), and capillary electrophoresis[13,14].

**TABLE 3**
**Amino Acid Content of Ethnic Hair Samples (μ*M*/g hair)**

| No. | Amino Acid | Africoid | Caucasoid | Mongoloid |
|---|---|---|---|---|
| 1 | Alanine | 44.3 | 45.3 | 45.9 |
| 2 | Arginine | 52.1 | 55.9 | 57.2 |
| 3 | Aspartic acid | 58.5 | 53.6 | 58.0 |
| 4 | Cystine | 3.3 | 2.3 | 3.0 |
| 5 | Glutamic acid | 150.0 | 139.0 | 143.0 |
| 6 | Glycine | 120.4 | 115.3 | 117.2 |
| 7 | Histidine | 56.2 | 59.1 | 56.5 |
| 8 | Isoleucine | 7.2 | 8.3 | 7.3 |
| 9 | Leucine | 23.3 | 20.8 | 24.4 |
| 10 | Lysine | 54.9 | 55.8 | 58.2 |
| 11 | Methionine | 16.9 | 23.7 | 18.7 |
| 12 | Phenylalanine | 1.7 | 4.6 | 2.6 |
| 13 | Prolyne | 13.9 | 14.8 | 14.4 |
| 14 | Serine | 74.6 | 76.2 | 72.0 |
| 15 | Threonine | 114.5 | 124.7 | 113.8 |
| 16 | Tryptophan | 68.4 | 67.6 | 67.2 |
| 17 | Tyrosine | 19.1 | 19.1 | 18.9 |
| 18 | Valine | 41.1 | 44.2 | 50.2 |

From Gold and Scriver[12].

Careful attention must be paid to the sample preparation. Before the dissolution of the hair, the sample should be washed in accordance with the procedure suggested by the IAEA[15], and then dried at room temperature and stored in a closed container.

Vermeir et al.[16] cleaned the hair sample with water and ethanol, ground it under liquid nitrogen to a final size down to 80 μm, and then homogenized in a drum. A solid sample of 100 mg was weighted into a digestion vessel and 1-ml sub-boiled concentrated nitric acid was added. The sealed vessel was placed into a microwave oven.

Irreversible solubility is characteristic for the dissolution of biological materials in solvents. After dissolution, the solute undergoes dissociation, decomposition, or reaction with the solvents.

More than a hundred pharmaceuticals, drugs of abuse, or doping agents have been reported to be detectable in human hair[13,17]. A number of drugs of abuse are unstable in the postmortem environment. This requires careful consideration when trying to interpret their significance. Heroin, morphine glucuronides, cocaine, and the benzodiazepines are particularly prone to degradation. The dissolution process and the solvent means that no concentration of a drug of abuse can be interpreted in isolation without a thorough examination of the relevant circumstances and relevant factors that could impact on the drug concentration corroborated[7,18].

Hair care products, such as shampoos, permanent wave solutions, and those for dying, bleaching, or shading, can disturb the concentration of fatty acid ethyl esters as markers of chronically elevated alcohol consumption. In particular, false-positive results were found for hair after its daily treatment with a hair lotion containing 62.5% ethanol, deodorant, or hair spray[19].

Regarding the performance of different hair testing laboratories, little information is available as to what extent test results are comparable and whether their interpretation is consistent[7].

Baptista et al.[20] dissolved a given mass of hair in nitric acid. The solution was then analyzed by the PIXE method for analysis of trace elements in human hair. HS-SPME coupled with the hydrolysis of the hair matrix by 4% sodium hydroxide (NaOH) in the presence of excess sodium sulfate ($Na_2SO_4$) has been used by Sporkert and Pragst[14] in hair analysis for organic compounds. The recoveries decreased in the case of drug solubility and dissolved hair proteins.

As described above, in addition to blood, urine, and saliva drug testing, hair samples are also used for an external drug test. Hair leaves a good trail since hair grows about 0.4 mm/day. Fifty millimeters of hair give approximately a 125-day history. Long hair can provide a drug test sample for a period covering 6–8 months.

*Note: Despite the highly developed drug-testing techniques, several pharmaceutical companies market powerful high-toxin-level drinks or super-concentrated herbal capsules and tablets as reliable masking products in order to pass a drug test. These products are made from natural herbal ingredients, including vitamins, minerals, and masking agents.*

Toxins in hair are part of the molecular structure and cannot be removed or cleaned. A portion of this molecular structure is made of the toxin. The toxins are stored in the fatty tissue of the skin. The body gives off skin oil or sweat, which releases toxins that will touch and be absorbed back into the hair. Molecules absorbed or transferred to the keratinized hair from perspiration (sweat, sebum, and transdermal excretion) represent the endogenous-exogenous pathway. Drugs deposited from the external environment (pollution, cosmetic hair treatments) can enter the keratinized hair by absorption and represent the exogenous pathway.

Most drugs are weak bases or acids that are present in biological fluids both in their ionized and nonionized forms.

The minimum detectable concentrations of drugs in hair have not been well established. The solvents most efficient in removing cocaine, for example, were 0.1 *M* HCl and dimethylformamide. However, even these solutions left approximately 30% of the externally applied cocaine in the hair.

The difference between decontamination (removal of exogenous drugs and other interfering substances) and extraction (removal of endogenous drugs for analysis) is often blurred. The two most prevalent types of extraction processes are

1. Digestion of the hair matrix and extraction of the drug from the digest with liquid-liquid or solid-phase extraction procedures, or
2. Extraction of the drug from the intact hair matrix.

The cocaine was incorporated into hair by exposure to a cocaine solution containing a radiotracer. After decontamination, the hair was extracted with 0.1 *M* HCl and the radioactivity in the HCl extract was measured. The hair was then digested with NaOH and the residual radioactivity measured.

A number of different decontamination procedures were tested to remove external contamination produced by solutions of drugs. None were sufficient to remove all drugs[9].

The results of the solubility determination of the hair samples were compared. These results were then compared with the results of the analysis using the same method of a reference hair sample furnished by the IAEA and the Reference Man data for the elemental composition of hair.

Garside and Goldberger[21] reported a good review of the analytical techniques utilized in hair analysis, including solubility and digestion of hair samples. The hair tests have been utilized in clinical, forensic, and epidemiological studies; historical research; and even presented as evidence in civil, criminal, and military courts of law.

A popular technique of hair analysis includes the washing of the hair sample, followed by cut or pulverizing, and incubation at 40ºC for about 24 h in a solution of 1 *M* NaOH or KOH and neutralizing with 0.1 *M* HCl before diluting further with pH 7, 1 *M* phosphate buffer. During incubation, a magnetic stir bar is used to provide constant agitation. Hair samples can be treated with a solution of 1 *M* NaOH and boiled until the hair disintegrates. The hydrolyzed sample is neutralized with 1 *M* HCl and analyzed directly.

There is no internationally accepted hair testing method, but there are some recommendations[9]. Most of the recommendations focus on drug testing in human hair.

In summary, hair samples have been washed, pulverized, and digested by several techniques including acidic (HCl, $H_2SO_4$, $HNO_3$) and basic (NaOH, KOH) hydrolysis, methanolic solvent, enzyme digestion, and supercritical fluid extraction[22,23].

Several investigators reported that different hair samples incorporate differing amounts of drugs when exposed under identical, *in vitro* conditions[24]. The uptake and retention of drugs by hair has at least four elements that may differ among various groups:

1. Permeability of the hair due to genetic influences
2. Use of cosmetic hair treatments
3. Removal of drugs during personal hygiene
4. Routine administration of drugs and passive exposure to drugs from the environment

For hair decontamination, a reported procedure involved washing with methanol, acetone, sodium dodecyl sulfate, isopropanol, dichloromethane, ethanol, and phosphate buffer.

These results strongly suggest that regardless of the decontamination procedure employed, external contamination cannot be fully removed. The mechanisms of drug accumulation in hair are unknown, and there is only limited information on the physicochemical characteristics of how drugs bind to hair. Once a drug is deposited in hair, it remains detectable for a period of months to years.

### Solubility

The concept of solubility in this series[1,2] has been described by two different approaches. According to the first scheme, the dissolved solute does not change its chemical structure in the solution, i.e., it is recoverable by unit operation, such as distillation, crystallization, etc. In the second scheme, the solute undergoes dissociation, decomposition, or reaction, and its chemical structure changes. When polymers or biological macromolecules are dissolved in a solvent, the solutes lose their original structure and cannot be recovered in their original form.

The first procedure is reversible, while the second is irreversible. The significant difference between the reversible and irreversible solubilities is the chemistry of the solute in the solution. If there is no interaction between the solute and solvent, then there is no change in the composition of the solute and the separation can be performed after the solubility.

Whether a biological material is soluble or insoluble in water is determined not only by its structure and the forces holding the constituted molecules together, but also by the melting point and the enthalpy of fusion of the solute. In addition, the binding forces between the solute molecules and those between the solvent molecules play a significant role.

The associated water molecules are bonded together by hydrogen bonds between the hydroxyl groups in the liquid state. It is expected that those substances will be soluble in water that can fit into the water structure and play the same or similar role as water molecules. Therefore, those organic substances that possess hydroxyl and/or carboxylic groups are water soluble.

As the solubility of hair in the reported solvents (acidic and alkaline) is irreversible, hair is not recoverable from the solution in its original form. The disintegrated/dissolved hair in the solutions is a jelly-like mass that could be diluted with water.

The solubility of higher hydrocarbons, alcohols, amines, etc., in water is very low. The heat of solution is generally small or even negative at room temperature. This is not expected in ideal, regular, etc., type solutions. The deviation is explained by the “iceberg” (i.e., more ordered) structure in the water surrounding the solute molecules[25,26].

Aqueous systems are very important in biological systems. In an aqueous environment, the nonpolar groups tend to adhere to one another, and this tendency has been referred to as hydrophobic bonding. However, the hydrophobic bonding has caused an enthalpy effect and the solubility in water will decrease by the iceberg formation.

In the dissolution of higher hydrocarbons in water, the destruction of hydrogen-bonding interactions and the formation of iceberg in the water surrounding the solute molecules are the dominating processes. The iceberg formation decreases with temperature rise and at approximately 160°C, the iceberg formation seems mostly to disappear. The solubility is much larger at lower temperature as compared with the hypothetical solubility assuming no iceberg formation.

The interaction between the solute and solvent molecules are of several types, such as:

- Electrostatic (dipole-dipole and ion-dipole)
- Dispersion forces
- Donor-acceptor
- Hydrogen bonding
- Structure breaking
- Structure making

The nature of solute-solvent interaction is responsible, for example, for the solvating of reactants and the transition state. Furthermore, in nucleophilic reactions, there is hydrogen bonding between a protonic solvent and the nucleophile (e.g., alcohols, water). When the nucleophile is uncharged, the hydrogen bonding is weak. The nucleophilic strength of carbon for halide ions is Cl<Br<I in water or alcohols, and the reverse in aprotic solvents. In protonic solvents, the hydrogen-bonding solvation is stronger for small nucleophiles. Solvents with dielectric constants greater than 15 (i.e., polar aprotic solvents) are capable of promoting nucleophilic reactions. Hydrocarbons, $CCl_4$ and so on, are nonpolar aprotic solvents.

If a polymer and solvent are mutually soluble, then the heat of solution per unit volume is small. Polymer solutions are characterized by large entropy of mixing in comparison with that for ordinary solutions.

Since the polymer molecule is much larger than that of the solvent, and it is a long chain, the free volume of the molecule becomes significantly larger, and this results in a large discrepancy between the observed and ideal cases. There are principally two decisive factors that dominate the mutual solubility between polymers and solvents: first, the hydrophile-lipophile balance of the molecules and second, a small change in temperature upon solution as a characteristic of polymers.

For mixtures of polymers and normal solvents, there is a strong negative deviation from Raoult's law. The phase equilibrium relations are described by the Flory-Huggins theory[27]. The theory is based on a lattice model that assumes that the polymer molecules are more densely packed than molecules of normal liquids. According to the theory, when the polymer solution is mixed with normal solvents, the polymer molecules gain greater freedom for vibrational and rotational motions; at the same time, the smaller solvent molecules partially lose such freedom.

The Flory-Higgins theory can be applied to multicomponent mixtures containing any number of polymers and any number of solvents. Limited solubility is often observed in solvent-polymer systems. The limited solubility ($\chi$) is given by the expression:

$$\chi > ½(1 + 1/m^{½})^2$$

where $m = V_2/V_1$, V = molar volume (polymer 2, solvent 1). For large m, the value of $\chi$ may not exceed ½ for miscibility in all proportions.

The introduction of a solute molecule into the solvent causes the formation of microscopic regions of weakness and expansion until enough energy has been absorbed by the system to restore the local pressure to the bulk internal pressure[28].

There are special problems with very sparingly soluble substances. The difficulty arises from three main sources:

1. Solubility is an equilibrium property that could be slow to establish.
2. Impurities affect the reliability of the results.

3. Heterogeneity in the energy content of the crystalline solids.

These difficulties can lead to large discrepancies in the reported values. The published aqueous solubility of cholesterol, for example, ranges from 0.025 to 2600 g/l[29].

There are several solvent properties that have been related directly or indirectly to the solubility. These parameters are molecular size (molar volume), molecular surface area, polarity, polarizability, H-bonding strength, and solubility parameter. Theory suggests that maximum solubility can be achieved when the solubility parameter of the solvent matches that of the solute. There is evidence that solvents whose n (refractive index), ε (dielectric constant), V (molar volume), and δ (solubility parameter) approach those of the solute are relatively stronger solvents. As a general rule, structural similarity favors solubility.

Keratin contains approximately 17% cystine:

$$HOOC\text{-}CH(NH_2)\text{-}CH_2S\text{-}S\text{-}CH_2CH(NH_2)\text{-}COOH$$

There are three important groups in cystine:

1. The carboxyl groups, which act in ester or salt formation
2. The amino groups, which are part of the peptide linkage through cross-chain formation by direct hydrogen bonding with other keratins
3. The sulfur to sulfur group, which is easily broken to form a gel-like mass in depilation

The breakdown of cystine is promoted by alkali ($OH^-$), sulfide ($S^{2-}$), or thiols (-SH), and the mixture of these ions.

Hair is one of the most difficult protein structures to digest, requiring, in addition to enzymes, such chemicals as dithiothreitol (reducing agent for proteins and enzymes) and detergents, i.e., chemicals that can degrade antibodies (defense proteins) even in the absence of enzymes (proteins to catalyze metabolic reactions), by disulfide bond cleavage and denaturation (unfolding the polypeptide chains of proteins).

Drug solubilization via digestion is universally applicable to all drugs, liberating these species quickly (within 1–6 h) from the protein matrix with 100% efficiency under mild conditions of temperature (25–35°C) and neutral pH[5].

There is a large variety in the workup and derivatization procedures for the detection of drugs in hair that has been reported[21,30]. During the dissolution procedure, it is very important to avoid degradation of the parent drugs. Among the most common dissolution procedures for hair is the incubation of hair in NaOH or KOH solution, and subsequently neutralizing with HCl at 80°C. The workup procedure contains an acidic, alkaline, or enzymatic hydrolysis. Some investigators from a round-robin study found that 0.1 *N* HCl solution is efficient in its removal of the target compounds from hair by enzymatic digestion that dissolves the hair.

Enzymes are formed in living cells. They are large molecules relative to substrates or functional groups they act upon. Enzymes are proteins formed from polypeptide chains, tightly folded into a compact globular form. There are over 2000 different known enzymes. Their molecular weights range from about 12,000 to over 1 million.

At the surface of the enzyme is a small patch or active site where the enzyme and substrate are bound to each other. In a biological reaction, one reactant or substrate forms a complex with the enzyme. After the enzyme-substrate complex formation, the substrate is altered by a series of transformations, resulting in a product that separates from the enzyme. A given enzyme can catalyze one particular biochemical reaction and no others.

Enzymes are greatly influenced by several factors, such as temperature, pH, activators (e.g., inorganic ions), and inhibitors (e.g., heavy metals; copper, mercury, etc.) that are poisonous and inhibit enzyme action completely. These regulatory materials activate or inhibit the enzyme action.

Keratinase is a water-soluble proteolytic enzyme that has the ability to digest the keratin in wool and other forms of hair, thereby converting a portion of it to water-soluble form. It thus acts as a depilatory and is used in removing hair from pelts and hides, as well as from human skin[31].

Hair solubility is influenced by amino acid composition and sequence, molecular weight, conformation, and content of polar and nonpolar groups in amino acids and their hydrophobic and hydrophilic groups. In addition, the solubility is also affected by the solvent types, e.g., aqueous, ionic, organic, etc. Particularly in ionic solutions, the solubility can increase (salting in) or decrease (salting out). The level of solubility decrease is affected by time and heating temperature.

Heating of hair above 60°C causes irreversible changes in protein structure that is also influenced by pH, ionic strength, and protein concentration. These changes result in protein denaturation and reduced solubility. The steaming of wool in a neutral or alkaline solution usually leads to a decrease in solubility. Dry heating causes the solubility to decrease, whereas oxidation or acid dyeing increases the solubility.

Exposed aromatic acids may play an important role in protein solubility, which is mostly related to the high hydrophobicity of aromatic amino acids. Protein solubility is significantly affected by the net charge on the protein molecules and is usually presented as a pH effect. Proteins tend to decrease in solubility as their hydrophobicities increase. The content of sulfhydryl groups has been related to the insolubilization of proteins.

During heat treatment, the insolubilization is caused by polymerization of the protein. However, when temperature increases, some protein structures may be unfolded to a straight chain, allowing more protein-water interactions that would increase protein solubility.

The solubility of protein could greatly increase at high temperature (about 120°C) in an alkaline media (above pH 11). The addition of 2% NaCl to aqueous protein solutions increases the number of charges on protein molecules and causes salting-in effects.

The protein insolubilization during drying is the result of protein polymerization by the disulfide bond interchange reaction with the increased number of SH groups. An increase in protein solubility can be obtained by protein depolymerization using reducing agents such as sodium bisulfite and cystine.

Acid-extracted protein showed poor solubility (about 50%) compared with alkali-extracted protein (about 90%).

Polyphenolic compounds react with peptide groups through hydrogen bonding at acid pH values and reduce the solubility of the protein.

High solubility in salt solutions is important for the practical use of most proteins. Protein solubility, in general, increases with increased pH in the range of pH 6.0 to 7.0.

There is evidence that ethnicity, hair color, and hair treatment affect the solubility of hair. Although there is no clear physicochemical or molecular basis for these differences in solubility between hair types, it is conceivable that differences in the structure and chemical composition of hair may affect the solubility of different hair types. However, whether or not variables such as hair treatments, hair color, and ethnicity affect the solubility of hair remains controversial. There are some suggestions that the solubility of hair may be affected by the similar variables that affect drug incorporation and trace element concentrations in hair.

The affinity of a solvent for hair is determined by the following molecular properties: size, the presence of polarizable groups, electric charges, and the ratio of hydrophobic to hydrophilic groups. Small molecules and molecules with hydrophobic groups appear to have higher affinity for hair.

There are some suggestions for selective solubility by particular hair types, but differences in structure, morphology, and protein structure between hair types may be the basis for the observed differences. However, there is definite need of further evaluation of the solvent and solubility testing procedure of particular hair types.

Several acidic or alkaline reagents have been proposed for dissolving hair samples, such as hydrochloric acid, sulfuric acid, nitric acid, perchloric acid, and NaOH solution[32]. Baptista and coworkers[20] dissolved 60 mg of human hair in 1 ml of pure nitric acid after careful preparation of the sample.

Human hair samples can be dissolved in 6 *M* HCl or 8 *M* $H_2SO_4$ solution within 1 h by heating at 90ºC. For rapid and efficient digestion, the scalp hair can be dissolved within 15 min in hot 10 *M* NaOH

solution[33]. In the procedure, about 0.1 g of the hair sample was digested in a 15-ml test tube with 0.2 ml 10 *M* NaOH over a hot bath at 90°C for 15 min. The digested sample solution was then analyzed for estimating body burden of organomercury components, thereby also providing an indirect measure of dietary intake.

Sporkert and Pragst[14] used 10 mg of washed and dried hair for dissolving in 1 ml of 1 *M* NaOH at 70–90°C during 30 min. Adding $Na_2SO_4$ to the solution caused a higher extraction yield than increasing the NaOH concentration to 30%.

The dissolution of the hair matrix in alkaline solution, e.g., 1 *M* NaOH, is one of the most efficient and convenient sample preparation methods if the samples are stable under dissolution conditions.

Knowledge of hair solubility can give useful information on the potential utilization of chemical depilatories. A depilatory is a substance used to remove hair from skin. On certain areas of the body, hair can be cosmetically unappealing. In the search for depilating agents, the pharmaceutical research focused on the removal of hair from the face, underarms, and legs.

A review of the various types of hair removal methods from the face and body has been reported by Liew[34]. Shaving, plucking, waxing, and chemical depilatories are effective in the short term, but not in the long term. The most commonly used chemical depilatories are mercaptens, particularly salts of thioglycolic acid, which work by hydrolyzing disulfide bonds. Hair strength is a function of the disulfide bonds between cystine molecules. Oxidizing agents, e.g., hydrogen peroxide and ammonium bicarbonate, reduce the appearance of unwanted hair by bleaching it; however, they soften the hair, but do not dissolve it.

Thioglycolates do not penetrate thick beard hairs rapidly enough to be a practical alternative to shaving. Instead, preparations based on barium, strontium, or calcium sulfides must be used. Sulfide depilatories are limited by the foul odors released by hydrogen sulfide gas[35].

The leather industry uses large amounts of sodium sulfide for removing hair from hides. The conventional process of preparation for depilation of skins for the manufacture of leather consists of the oxidation of the hair by chlorine dioxide in acid medium. The dissolution of the hair is due to the rupture of the disulfide bridges and to the formation of sulfonic groups along the hair protein portions. An alternative process involves the oxidation of the hair by the $H^+/Cr_20_7^{2-}$ couple in an oxidizing bath consisting of sodium or potassium dichromate in strong mineral acid, e.g., hydrochloric acid, nitric acid, sulfuric acid, perchloric acid, or a mixture of acids. Skins are immersed in the bath under continuous agitation at 20–25°C. At the end of 24 h, the hair forms a sludge dispersed in the bath[36].

Cystine is found in greater quantities in hair (15% of the keratin) vs. in skin keratin (2%), thus primarily targeting the structural integrity of hair over epidermis. This explains why thioglycolates preferentially hydrolyze the keratin in hair over that in skin. The added alkali (e.g., NaOH) increases the pH and the efficacy of the thioglycolate. However, these agents may cause irritation on sensitive areas. Boric acid solution is recommended to counteract the effects of the alkalinity.

Depilatories act in the follicle where the hair shaft has not fully keratinized and therefore can rapidly absorb the chemical. Chemical depilatories are prepared in aqueous solutions of thioglycolic acid mixed with alkali, such as NaOH or calcium hydroxide. The concentrations of the chemical depilatories vary depending on the surface hair of the body[37]. The auxiliary ingredients of typical depilatory creams have been reported by Webber[38] (see Table 4). The concentrations of commercially marketed depilatory creams are also listed in Table 4. The dominating active ingredient in most depilatories is calcium thioglycolate and the pH values range between 10 and 12.5.

A large number of chemical depilatories are listed in the patent literature[39,40]. The commercially marketed chemical depilatories are available from several manufacturers as aerosol sprays, gels, pastes, creams, or lotions[6,38]. The application of a depilatory takes between 3 and 15 min. The dissolved hair shaft becomes a jelly-like mass, which is easily wiped away with a washcloth. For the removal of undesirable hair from sensitive skin, a Nair® cream has been recommended by Wendelin et al.[41]. Nair® contains calcium thioglycolate and sodium thioglycolate, which dissolve hair by breaking the disulfide bonds between cystine molecules. Thioglycolate cream penetrates into the follicle, thereby removing hair below the skin surface. The composition of Nair® is listed in Table 5[42].

**TABLE 4**
**Ingredients of Depilatory Creams**

| Ingredients | Concentration (%) |
|---|---|
| Calcium thioglycolate trihydrate | 6.0 |
| Calcium carbonate, light, USP | 21.0 |
| Calcium hydroxide, USP | 1.5 |
| Cetyl alcohol, flakes, NF | 4.5 |
| Sodium lauryl sulfate, USP | 0.5 |
| Sodium silicate solution, 42.5° Bé | 3.5 |
| Perfume | 0.5 |
| Distilled or deionized water | 62.5 |
| Total | 100.0 |

Examples:

"Nair Brush-on Facial Hair Remover" (Church & Dwight Co., Inc.)

Aqua, Paraffinum liquidum, Calcinno thioglycolate, Cetearyl alcohol, Calcium hydroxide, Sodium hydroxide, Ceteth-20, Glycerin, Gossypium, Parfum, Xanthan gum, Phenoxy ethanol, Methylparaben, Butylparaben, Ethylparaben, Propylparaben, Isobutylparaben, Amyl cinnamal, Benzyl benzoate, Benzyl salicylate, Butylphenyl, Methylpropional, Linalool.

"Facial Hair Removing Cream" (The Boots Company PLC)

Aqua, Urea, Dihydrogenated tallow methylamine, Cetyl alcohol, Potassium thioglycolate, Butyrospermum parkii, Paraffinum liquidum, Ceteth-20, Polysorbate 20, Sodium magnesium silicate, Calcium hydroxide, Parfum, Steareth-10, Sodium gluceptate, Tetrasodium pyrophosphate, Propylene glycol, Aloe barbadensis, Chamomilla recutita, Phenoxyethanol, Methylparaben, Ethylparaben, Propylparaben, Butylparaben, Isobutylparaben, Cl 77891.

**TABLE 5**
**Ingredients of Nair® Cream**

| Ingredients | Concentration (%) |
|---|---|
| Calcium thioglycolate | 8.0 |
| Water | 75.0 |
| Unspecified alkaline material | 4.5 |
| Emulsifier | 1.0 |
| Waxes | 4.0 |
| Humectant | 7.5 |
| Some perfume | — |
| Total | 100.0 |

From Douglas[42].

In addition to cosmetic treatment, depilatory substances may be used when entrapped hair is tightly wound around an infant's finger or tightly wrapped around the toes. In a reported case, hair could not be removed without cutting the edematous skin. After the application of the hair remover, the strands of the hair either ruptured or dissolved so that they could easily be unwound from the finger[42].

### RESULTS

A conventional method of measuring solubility is the introduction of an excess amount of the material of interest (usually in pulverized form) into a flask containing the solvent (e.g., water) and stirring or shaking the flask for a number of hours until saturation is reached. After filtration or centrifugation, the concentration of the solute in the solvent is measured with an appropriate analytical technique, depending on the nature of the material[43].

Most solubilities are reported in units of mg/l or the equivalent $g/m^3$, although units such as mass percent and mole fractions have been used. The use of percent, ppm, ppb, or ppt should be avoided because of possible ambiguities.

For most of the structurally complicated materials, either no solubility data were available or the information was of a purely qualitative nature; for example, a statement to the effect that the material was "relatively insoluble".

A review of the various solvents used for dissolving/digesting hair of different types is listed in Table 6. In general, the solubility/digestion methods used in animal (sheep, mice, guinea pigs, and rats) hair are similar to those found for human hair[44].

Information on solubility of materials can be expressed in various forms: soluble, insoluble, slightly soluble, approximate solubility values, etc. All these phrases refer to the qualitative solubility. For a quantitative measurement of the solubility, the results are expressed by the weight of the solute (pulverized hair) dissolved in the specified volume of the liquid solvent (e.g., alcohol).

All data listed in Table 6 are qualitative solubility values. Solubility data for hair have not been found in the available technical and medical literature. Probable reason for the lack of quantitative data is the practical and commercial application of the experimental solubility values. It is likely that similar reasons can be assumed for the lack of quantitative solubility data for most of the structurally complicated materials.

For the application of structurally complicated materials, it is often sufficient to find solvents for dissolving the solutes partly or completely at the specified condition and quantitative measurement is not required. A typical example is the dissolution of dry paints on metal surfaces or removal of burned food from a frying pan by suitable solvents. For both cases, the information on the qualitative solubility in the solvents is needed.

### COMMENTS

The information on the solubility of structurally complicated materials in various solvents is mostly in the qualitative form. Quantitative solubility data, given in weight of solute (e.g., milligrams, grams, etc.) per volume (milliliter, liter, etc.) of the solvent were not found in the available literature.

Many of the body parts, e.g., bone, hair, skin, etc., belong to the structurally complicated materials without solubility data.

The determination of the solubility of a structurally complicated material in a solvent is outlined above. Conventional laboratories have the required equipment for performing the experiments. The cost of the experimental work may not be expensive.

One may ask the question, Why are experimental solubility data for structurally complicated materials not published? Probably the main reason could be the lack of need for scientific and commercial application at the present time. This should change in the near future.

**TABLE 6**
**Solubility/Digestion of Hair in Solvents**

| Type of Hair | Solvent | Temp. (ºC) | Time | Comments | Ref. |
|---|---|---|---|---|---|
| Head | 4% NaOH + $Na_2SO_4$ | 70–90 | 30 min | Male and female | [14] |
| Human | 70% $HNO_3$ | Hot | n.sp. | n.sp. | [4] |
| Human | cc.$HNO_3$ | Sub-boiled | n.sp. | Ground to 80 µm | [16] |
| Head | 0.1 *N* HCl | 45 | 24 h | Powdered | [45] |
| Scalp | Pure $HNO_3$ | n.sp. | n.sp. | Added $Sr(N0_3)_2$ | [20] |
| Head | 10 *M* NaOH | 90 | 15 min | Analysis of hair | [33] |
| Scalp | 3 *N* HCl | 40 | Overnight | Comparability | [46] |
| Scalp | 30 $H_2SO_4$ /70 $HNO_3$ | n.sp. | n.sp | Mercury analysis | [47] |
| Cattle | 10% TMAH | n.sp. | n.sp. | Solubility of hair shaft | [48] |
| Scalp | 1 *N* $NH_3$ | n.sp. | n.sp | Extractable protein | [49] |
| Guinea pigs | 1 *N* NaOH | 37 | 24 min | Cross-linkage in proteins | [50] |
| Scalp | 0.1 *M* HCl or $H_2S0_4$ | 45 | Overnight | Pulverized | [44] |
| Scalp | Proteinase K solution | 40 | Overnight | In digest buffer | [44] |
| Scalp | 1 *N* NaOH | 100 | 60 min | Degrades cocaine | [44] |
| Scalp | 0.1 *M* HCl | 37 | 18 h | Unpulverized | [51] |
| Scalp | 0.05 *M* $H_2SO_4$ | n.sp. | n.sp. | Extraction | [52] |
| Mice | $CH_3OH$ extraction | 60 | 60 | 2-h Extraction | [53] |
| Head | 0.1 *M* HCl | 45 | 24 h | Extraction | [54] |
| Head | 10 ml $H_2SO_4$ + 15 ml $HNO_3$ + 1 g $K_2MO_4$ | Room | 2 h | Spectrometry | [55] |
| Head | 1 ml $HNO_3$ | n.sp. | n.sp. | PIXE method | [56] |
| Head | 2 ml $H_2SO_4$ + 5 ml $HNO_3$ | 140 | 40 min | Severity test | [57] |
| Wool | 0.1 *N* NaOH | 65 | 60 min | Pulverized | [58] |
| Wool | 0.1 *M* HCl | 45 | Overnight | Pulverized | [58] |
| Wool | Glucuronidase-aryl-sulfate phosphate buffer | 40 | 2 h | Enzymatically digested | [59] |
| Rat | Sodium dodecyl sulfate | n.sp. | n.sp. | Solubilized | [60] |
| Rat | β-Mercaptoethanol | n.sp. | n.sp. | Solubilized | [60] |
| Wool | 25% $(NH_2)_2CO.HSO_3$ | 65 | 45 min | Gravimetric | [43] |

### ACKNOWLEDGMENTS

The author is indebted to Imperial Chemical Industries for providing the facilities and support of this project. Thanks are also due to a number of colleagues at ICI for helpful discussions and their welcome suggestions. Finally, I want to thank my wife Joan, who checked the text and helped with the whole enterprise.
